# Biodiversity of Pigmented Fungi Isolated from Marine Environment in La Réunion Island, Indian Ocean: New Resources for Colored Metabolites

**DOI:** 10.3390/jof3030036

**Published:** 2017-07-02

**Authors:** Mireille Fouillaud, Mekala Venkatachalam, Melissa Llorente, Helene Magalon, Pascale Cuet, Laurent Dufossé

**Affiliations:** 1 Laboratoire de Chimie des Substances Naturelles et des Sciences des Aliments—LCSNSA EA 2212, Université de La Réunion, 15 Avenue René Cassin, CS 92003, F-97744 Saint-Denis CEDEX 9, Ile de La Réunion, France; mekalavenkat@gmail.com (M.V.); melissa.llorente@gmail.com (M.L.); laurent.dufosse@univ-reunion.fr (L.D.); 2 Ecole Supérieure d’Ingénieurs Réunion Océan Indien—ESIROI, 2 Rue Joseph Wetzell, F-97490 Sainte-Clotilde, Ile de La Réunion, France; 3 UMR ENTROPIE and LabEx CORAIL, Université de La Réunion, 15 Avenue René Cassin, CS 92003, F-97744 Saint-Denis CEDEX 9, Ile de La Réunion, France; helene.magalon@univ-reunion.fr (H.M.); Pascale.cuet@univ-reunion.fr (P.C.)

**Keywords:** fungi, biodiversity, Indian Ocean, Marine, coral reef, genotyping, pigment production

## Abstract

Marine ecosystems cover about 70% of the planet surface and are still an underexploited source of useful metabolites. Among microbes, filamentous fungi are captivating organisms used for the production of many chemical classes of secondary metabolites bound to be used in various fields of industrial application. The present study was focused on the collection, isolation, screening and genotyping of pigmented filamentous fungi isolated from tropical marine environments around La Réunion Island, Indian Ocean. About 150 micromycetes were revived and isolated from 14 marine samples (sediments, living corals, coral rubble, sea water and hard substrates) collected in four different locations. Forty-two colored fungal isolates belonging to 16 families, 25 genera and 31 species were further studied depending on their ability to produce pigments and thus subjected to molecular identification. From gene sequence analysis, the most frequently identified colored fungi belong to the widespread *Penicillium, Talaromyces* and *Aspergillus* genera in the family Trichocomaceae (11 species), then followed by the family Hypocreaceae (three species). This study demonstrates that marine biotopes in La Réunion Island, Indian Ocean, from coral reefs to underwater slopes of this volcanic island, shelter numerous species of micromycetes, from common or uncommon genera. This unstudied biodiversity comes along with the ability for some fungal marine inhabitants, to produce a range of pigments and hues.

## 1. Introduction

With the growing demand for natural compounds in the industrial sector, marine derived fungi appear to present many interests. Filamentous fungi are ubiquitous in nature due to their huge capacity of adaptation and their ability to produce an assortment of new secondary metabolites. Literature now abundantly reports the significant involvement of fungi in the industry, through the production of various useful substances, such as antibiotics, immunosuppressants, anti-cancer drugs, plant hormones, enzymes, acids and also natural pigments [1,2,3,4,5]. Both the pigments and enzymes equally find their usages in food and beverages, animal feeds, pharmaceuticals, cosmetics, textile, leather, pulp and paper industries, biofuel production, and environment bioremediation [6].

Nevertheless, the distribution of the marine-derived fungal species and their contribution to marine biotopes are still in infancy, and more has to be explored [7,8,9,10,11]. The highest diversity of marine fungi seems to appear in tropical regions, mainly in tropical mangroves, which are extensively studied because of their high richness in organic matters and especially lignocellulosic materials, favorable to the development of a wide range of heterotrophic microorganisms [11,12,13,14]. Anyway, many marine ecological niches are still unexplored and it seems plausible that unique features of marine environments can be the inducers of unique substances, biosynthesized by marine or marine-derived microorganisms [15,16].

Considering the immense genetic and biochemical diversity of these fungi, partially derived from the specificity of the biotopes they are facing, marine-derived fungi are regarded as a potential bright source of new molecules with likely application in pigment production [17,18]. Many genera producing pigments have then been isolated either from water, sediments, and decaying organic residues, or from living organisms such as invertebrates, plants or algae. Fungi belonging to genera such as *Aspergillus, Penicillium*, *Paecilomyces*, *Eurotium*, *Alternaria*, *Fusarium*, *Halorosellinia*, *Monodictys* and *Microsphaerospsis* have already been identified from marine biotopes [19,20,21]*.* They are therefore able to exhibit bright colors, from yellow to black, mainly belonging to polyketides. Indeed, polyketides pigments and particularly azaphilones and anthraquinones seem to dominate marine natural products of fungal origin [22]. Colored compounds, usually described as secondary metabolites, do not seem to be directly involved in the primary growth of the fungus in which they occur [23]. However they may play some important roles in the resistance to a variety of adverse environmental factors (desiccation, exposure at extreme temperatures, irradiations and photo-oxidation) or in ecological interactions with other organisms (macroorganisms such as sponges, corals or other microbial communities) [24]. For this reason, many fungal secondary metabolites exhibit useful biological activities and are of interest to the pharmaceutical, food, and agrochemical industries [16,25].

This study initiated the search for filamentous fungi in some tropical marine biotopes of coral reefs and underwater slopes of the volcano from La Réunion Island. Fungal isolates from samples of sediments, seawater, hard substrates, coral rubbles or living coral individuals (*Pocillopora* sp.) were characterized both by phenotypic and molecular ways. The production of pigments of quinoid-type produced from the mycelia cultured in liquid media was used as a first approach to screen for the pigment production. This work reveals a part of the mycofloral biodiversity in La Réunion Island tropical marine environment and its potentiality to propose new pigment sources to expand in an industrial setting.

## 2. Materials and Methods

### 2.1. Samples Collection

**Figure 1:**
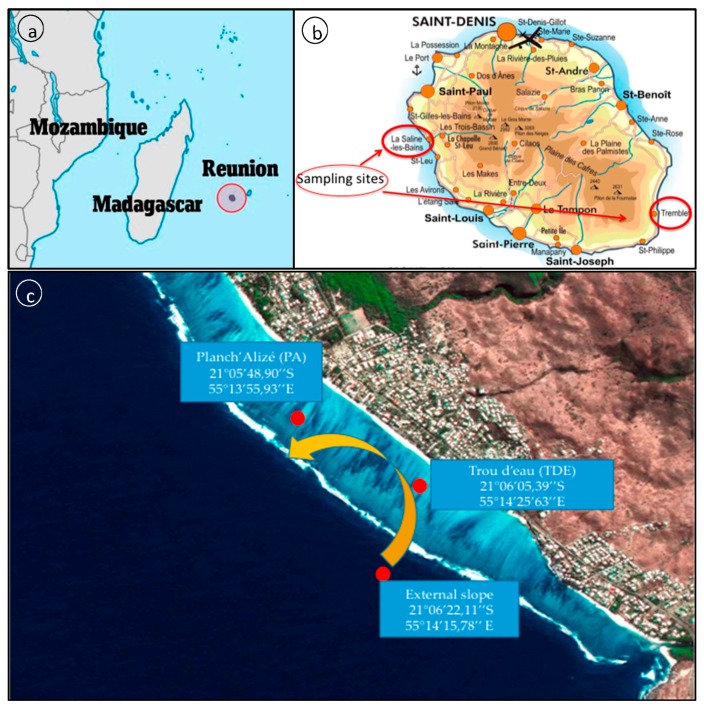
(**a**) La Réunion island location (Indian Ocean, 21°06′54.5′′ S and 55°32′11.0′′ E); (**b**) geolocation of sampling sites around La Réunion Island (West: La Saline; and East: Sainte Rose and Tremblet); and (**c**) geolocation of the three sampling spots at La Saline fringing reef: Trou d’eau (TDE inner reef and TDE outer slope ) and Planch’Alizé (PA) (back arrow represents the main water flow).

La Réunion Island lies in the Indian Ocean and is located 800 km east of Madagascar (21°06′54.5′′ S and 55°32′11.0′′ E) (Figure 1a). This tropical island arose two million years ago from a volcanic hot spot (Piton de La Fournaise) and is known for its rainforested interior and its fringing reefs holding most of the marine wealth.

A first set of samples was collected on the fringing reef from La Saline, which lies on the dry west coast of the island. It is more than 9 km long and ranges in width from 50 m in its northern part to 600 m in the south [26]. For the purpose of research, samples were collected from three sampling spots on the west coast that cover the sites of Trou d’Eau (TDE) (inner reef flat at −1 m depth and outer slope at −17 m) and Planch’ Alizé (PA) (inner reef flat, −1 m) (Figure 1b,c). Planch’ Alizé is considered as a sheltered site, located downstream of seawater flowing over the Trou d’Eau (Figure 1c). The outer slope is found at the outer edge of the reef, closest to the open ocean, and is characterized by spurs and grooves extending downward to the sand bottom, while the inner reef flat displays wide transversal strips of branched coral colonies alternating with narrow detrital channels perpendicular to the reef flat [27,28,29,30,31]. Low water flow and high solar radiation contribute to heating the reef water during the day, inducing important daily sea surface temperature variations. This area is also heavily laden with organic and mineral matter coming from nearby human activity (seaside area).

A second set of samples was collected in Sainte Rose area (south-east) on the submerged lava flows (Figure 1b). Indeed, the Piton de la Fournaise is one of the most active effusive volcanoes in the world with 27 eruptions between 1998 and 2007 and a mean frequency, over a century, of an eruptive phase every nine months. Submerged lava flows appear on the south-east part of the island when, during eruptions, the pool of lava overflows the active volcano mouth and pours down on the slopes of the volcano, to the sea. These costal marine ecosystems facing the deep ocean, are then regularly subjected to natural hazards such as being covered by incandescent lava flows, temporary changes in physicochemical conditions of water bodies and exceptional rises of temperature. Besides, this area is poorly inhabited and urbanized and, as a consequence, the amount of organic matter poured in the sea is reduced compared to other coastal ecosystems. It provides a natural laboratory to study the colonization of a blank substrate and the evolution of the biodiversity all around, during the following years. Samples were then obtained from sediments extracted from 1977 lava flow (−25 m depth) and 2004 lava flow (−70 m), as well as from surrounding free water at −70 m.

Seawater, sediments, parts of living corals and hard substrates (volcanic rocks or coral rubbles) were collected in sterile bottles, during the months of April and May 2012, stored in a cooling box (4 °C), brought to the laboratory, and treated immediately for the fungal isolation.

### 2.2. Culture and Purification of Fungi

To cultivate the revivable fungi from the collected seawater, 100 mL of water was filtered using a 0.45 µm sterile cellulose-nitrate filter (Sartorius Stedim, Göttingen, Germany). The filters were then placed in Petri plates containing malt extract agar (MEA) and Sabouraud agar (BD Difco, Franklin Lakes, NJ, USA) prepared with natural seawater collected near La Saline, and beforehand sterilized at 121 °C, 15 min.

The other samples such as sediments, hard substrates and parts of living/dead coral were treated separately. The samples were first washed with 70% alcohol and rinsed in sterile seawater. Then they were ground using sterile pestle and mortar. Ground material (5 g) was taken from each sample and added to 15 mL of sterile diluent (1.6 g of tryptone (Sigma- Aldrich, T-9410, Saint Louis, MO, USA), 0.05 g of tween 20, 1 L of sterile seawater of pH = 7.5). After stirring for 20 min at 150 rpm on a shaking table (Edmunt Bühler GmbH, VKS 75 Control, Hechningen, Germany), the suspension was diluted by employing serial decimal dilution method up to 10^−3^ [32]. Each diluted sample (1 mL) was poured on Petri plates containing MEA and Sabouraud agar prepared with natural seawater.

All the platings were performed in triplicates and incubated at 25 °C for 21 days. During this period, the plates were checked each day for the appearance of new colonies. Each new colony was individually isolated and cultured on new MEA solid medium. During the growth period, the production of colors was observed.

All the isolated fungi were cultured using monospore technique for future experiments and long-term storage. The fungi grown after 5 days were scraped and transferred into a sterile vial containing a cryoprotectant medium composed of 15% skimmed milk and 2% glycerol for long term storage at −80 °C [33,34]. In total, 42 fungal isolates were then selected for pigment production based on the visual appearance of the thalli grown on solid culture media.

### 2.3. Fungal Identification

#### 2.3.1. Fungal DNA Extraction

To extract DNA from the 42 purified isolates, a small amount of mycelium along with spores was cultivated on potato dextrose agar (PDA) at 25 °C under day light exposure. After 5 days of growth, the fungal mycelium was scraped and DNA was extracted using DNeasy Blood and Tissue kit (Qiagen, Hilden, Germany) following the manufacturer’s instructions. DNA amount and purity contained in each extract were evaluated by measuring the absorbances at 230, 260 and 280 nm (Nanodrop 2000, Thermo Scientific, Waltham, MA, USA) and calculating the ratio A_260_/A_280_ and A_260_/A_230_. DNAs were stored at −20 °C prior to use for amplification studies [35].

#### 2.3.2. Primers, PCR Amplification and Sequencing

The choice of PCR primers was made based on observed phenotypic characteristics for molecular identification. *Aspergillus* species were amplified for calmodulin gene using primers Cmd5/Cmd6 and *Penicillium* species for β-tubulin using primers T10/Bt2b [36]. To amplify and sequence the DNA from *Trichoderma* and *Hypocreales* species, EF-1H/EF-2T primer pair was used to amplify a fragment of the translation elongation factor 1 alpha gene (*Tef1*) [37]. For uncharacterized fungi, the fragments containing ITS region were amplified using ITS1-F_KYO2/ITS2 or ITS3_KYO2/ITS4, and, when necessary, the large subunit rDNA was also amplified using V9G/LR3 primer pair (Table 1) [36,37,38].

PCR reactions were carried out in a total volume of 30 µL: 1× of MasterMix (Applied Biosystems, Foster city, CA, USA), 0.5 μM of forward and reverse primers and at least 1.3 ng/μL of genomic DNA. Amplifications were carried out on a thermal cycler GeneAmp^®^ PCR System 9700 (Applied Biosystems) according to the following program: 94 °C for 5 min + 35 × (94 °C for 30 s, 55 °C (or 52 °C for the primers of calmodulin: Cmd5/Cmd6) for 60 s, 72 °C for 60 s) + 72 °C for 5 min for final elongation step.

#### 2.3.3. Sequence Analysis

Amplicons were sequenced in both directions (GENOSCREEN, Lille, France). The obtained electropherograms were read and corrected with Chromas software (version 2.13, Technelysium pty Ltd., South Brisbane, Australia). The extracted sequences for each gene were separately used to perform nucleotide searches using online BLAST algorithm, provided by NCBI (http://www.ncbi.nlm.nih.gov/BLAST/). BLAST results were sorted based on the maximum identity to the query sequence and considered as the best hit. Sequence-based identities with a cutoff of 97% or above and query coverage >90% were considered as significant [40,41]. Because of low recovery rates and concordance values, some isolates were amplified and sequenced a second time, with additional sets of primers, mainly among the isolates of the genera *Penicillium* and *Trichoderma*.

### 2.4. Culture Conditions for Pigment Production and Separation of Biomass from Liquid Medium

#### 2.4.1. Culture Conditions

Erlenmeyer flasks (250 mL) containing 80 mL of potato dextrose broth (PDB) medium were autoclaved at 121 °C for 15 min. Then, 120 mg of mycelia from interesting fungal species grown on PDA Petri plates were transferred into the sterile flasks and incubated at 25 °C under daylight exposure, with an agitation of 150 rpm for 10 days (Multitron Pro, Infors HT, Bottmingen, Switzerland).

#### 2.4.2. Separation of Biomass from Culture Liquid

After the end of the fermentation period, the culture medium containing extracellular pigments was separated from mycelia by vacuum filtration using Whatman filter paper No. 2 (Merck, Darmstadt, Germany). Thus, liquid medium and biomass were treated separately. The wet mycelium was further used for the extraction of pigment content.

### 2.5. Production of Pigments

#### 2.5.1. Determination of Pigments Production in Liquid Cultures

Chromophore is a chemical group that absorbs light of specific frequency and confers color to a molecule. Widespread polyketide pigments such as anthraquinones or azaphilones are often highly substituted aromatic molecules, with fused benzene rings [42]. Thus the majority of the common chromophores from fungi absorb in the UV region (one or several peaks between 200–300 nm), whereas absorbance in the visible region (400–700 nm) highly depends on the nature and the number of substituted groups.

To compare the pigment production of all isolates cultured in PDB medium, the amount of pigments produced in liquids was expressed as mg equivalent (mg eq.) of a chosen commercial standard per liter of culture medium (mg eq. purpurin L^−1^). Purpurin was chosen as a polyketide pigment in orange-red hue, which absorbs in the UV area (250–270 nm) as many polyketides [43], and also in the visible range 458–520 nm [44]. Thus, the absorbance of an authentic colored standard purpurin (Sigma-Aldrich) was estimated at different concentrations using an UV-visible spectrophotometer (Shimadzu UV-1800 Spectrophotometer). Then, in regard with the diversity of pigments content in the fungal cultures and as a preliminary approach, the absorbance of each sample was measured at 254 nm and the amount of pigments produced was expressed in “mg equivalent purpurin L^−1^” (Figure S1). In addition, for each isolate, the intracellular (IC) pigments (extracted from the biomass) and extracellular (EC) contents (liquid from culture, separated from the biomass) were scanned between 200 and 600 nm with a UV-1800 spectrophotometer (Shimadzu UV spectrophotometer, Shimadzu Corporation, Kyoto, Japan) in a quartz cell of 10 mm path length.

#### 2.5.2. Extraction of Pigments

IC pigments contained in the wet mycelium were extracted using a methanol: water combination (1:1 *v*/*v*) as conventional extraction method. The mixture was immersed in an ultrasonic bath at 45 °C for 30 min. The suspension was allowed to stir overnight at room temperature on a shaking table (VKS 75 Control, Edmunt Bühler GmbH). On the following day, it was filtered through Whatman filter paper No. 2 to recover the solvent containing the pigments extracted from biomass.

To compare the amount of pigments produced within the cells with the one diffused into the extracellular medium, we performed the nonparametric Mann–Whitney–Wilcoxon test as our data did not follow the normal distribution using the R software (R Development Core Team 2016) [45].

## 3. Results

### 3.1. Diversity of Isolated Fungi

More than 150 isolates were first recovered from the 14 samples collected among four locations. Among them, 42 were selected for identification, according to their capacity to develop colored mycelia or to secrete colored compounds in the media.

After sequencing, the 42 colored isolates were assigned to 16 families, 25 genera and 31 species (accession numbers mentioned in Table 2). The vast majority of the isolates have been identified with more than 98% concordance rate and recovered with high precision at the species level. However, few fungi (*Acremonium* sp., *Periconia* spp. and *Biscogniauxia* sp.) were identified to the genus level only, according to the gene chosen. The genetic characterization with these primers partially failed for two isolates (*Whalleya microplaca B* and *Wallemia sebi*). *Wallemia sebi* was only characterized according to morphological criteria.

Pigment producing fungi (42) were isolated from all types of samples: sediments (3), living coral *Pocillopora* sp. (7), unidentified coral rubbles (4), hard substrates (reef basis or volcanic rocks) (13) and seawater (15) (Table 2).

The most represented fungi, in the selection of colored micromycetes, belonged to the family Trichocomaceae with *Penicillium, Talaromyces* and *Aspergillus* genera (11 species); and then came the Hypocreaceae with *Trichoderma, Hypocrea* and *Acremonium.*

A high diversity of pigmented isolates was observed from the so-called “hard substrates” (rocky basis on which the coral colonies recruit, or submerged lava flows). Some *Nigrospora*, *Sporisorium*, *Whalleya*, and *Rhodotorula* isolates were collected from the outer slope at Trou d’Eau (TDE), although they are rather rarely isolated from marine environment. In Sainte Rose, *Penicillium* species (*P*. *citrinum*, *P*. *viticola*) as well as *Fusarium equiseti*, *Epicoccum sorghi*, *Nectria haematococca*, were successfully revived from lava flow, sampled at −25 m.

From our study, the coral rubbles (dead parts of corals) contained colored *Penicillium* or related species: *P. herquei* and two isolates of *Talaromyces albobiverticillius* (B and C), as well as an isolate of *Chaetomium globosum*. Coral rubbles or hard substrates naturally appear diversely colored underwater. Indeed, they support the colonization by multiple organisms (colored algae or other aquatic organisms), visually detectable when sampling.

The revivable colored fungi sheltered by the living coral *Pocillopora* sp. belonged to the genera *Aspergillii* (*A. creber*, *A. sydowii* and *Eurotium amstellodami* (the teleomorphic form of *A. amstellodami*), as well as to *Penicillium* (*P. viticola*), *Hypocrea (H. koningii*) and *Acremonium*.

Some fungal species were identified from different types of samples in the same area. At TDE outer slope, *Talaromyces albobiverticillius* A came from sediment and *T. albobiverticillius* B and C were revived from coral rubbles. *Nectria haematococca* was found in lava substrate (−25 m) (isolate B) as well as seawater (−70 m near lava flow) in the same area (isolate A).

Some similar species also appeared in separate locations: *Aspergillus sydowii* was found near lava flow on the east coast (seawater, −70 m) (isolate B) and also in living *Pocillopora* colonies (isolate A), from the west coast back reef (PA site). *Penicillium viticola* was isolated from the west coast on living *Pocillopora* sp. coral in PA (isolate C), from seawater in TDE back reef (isolate B), as well as from lava flow hard substrate (−25 m), on the east coast (isolate A).

These fungi found in several samples and/or in different locations may be considered as frequent in this marine environment.

### 3.2. Pigment Production

#### 3.2.1. In Culture Broth

**Figure 2:**
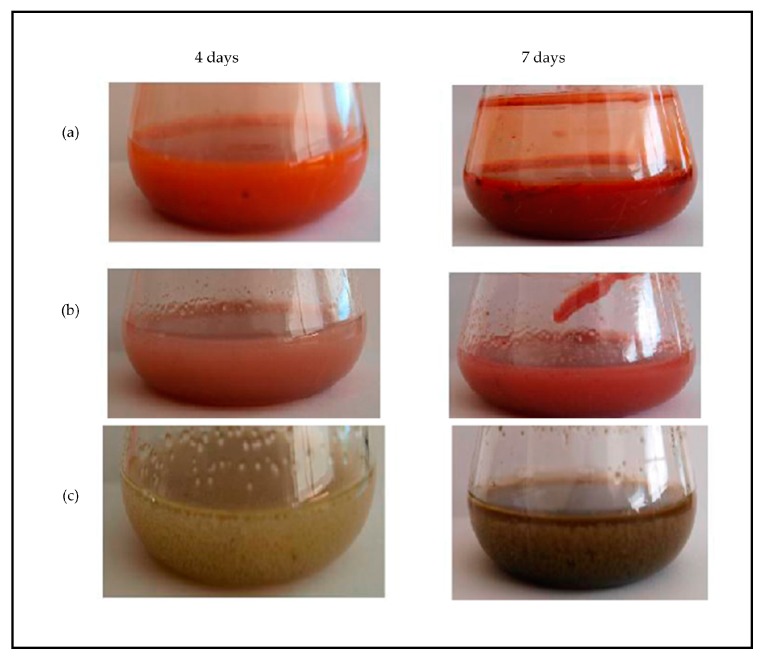
Colors observed in potato dextrose broth cultures from (a) *Talaromyces albobiverticillius* B, (b) *T. albobiverticillius* C, and (c) *Aspergillus creber* B, after four and seven days.

The majority of the isolates produced pigments after four days of fermentation in PDB. The colors of the broth (biomass plus liquid culture medium) always darkened over time, which indicated their potential for pigment production (Figure 2).

Overall, it was observed that the coloring trend was not directly related to the genus. Even if dominant colors such as yellow, red, brown, purple, orange, pink and green were observed in flasks, the hues were extremely diverse according to the species, even to the isolates (Table 3).

**Figure 3:**
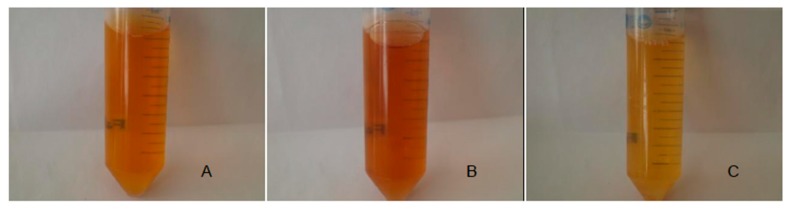
Colors observed from culture filtrates from three isolates *of Penicillium viticola* (**A**–**C**) (seven-day cultures in potato dextrose broth).

Indeed, some similar-looking fungi, identified under a unique accession number (i.e., sharing the same sequence for the considered gene), nevertheless developed different color-phenotypes, while cultured under the same culture conditions. As an example *A. creber* A developed a red hue, clearly different from the green-like color *of A. creber* B (“broth” column in Table 3). Moreover, if the same coloring trend was applicable to all the three isolates of *T. albobiverticillius* (A–C) or *P. viticola* (A–C), different shades of red or yellow-orange hues, respectively, were noticed (Figure 3).

Oppositely, no clear difference could be visually established among the pale pink shades of the three isolates of *F. equisetti* (A–C) or the two *N. haematococca* isolates (A and B).

#### 3.2.2. Pigmented Contents from Mycelium

For all pigment-producing isolates, the intracellular pigments (from mycelium) were extracted from the biomass. The approximate colors visualized after extraction are presented in Table 3. The pigments from most of the extracellular fungal culture filtrates were of dominating red, orange, yellow, green, brown, pink and violet. However, after extraction from biomass, many intracellular samples were uncolored, especially for isolates producing extracellular culture filtrates of pink, yellow and green color. This is probably characteristic of isolates essentially secreting water-soluble colored molecules in the culture media.

Instead, many dark colored cultures, mainly in the shades of red or maroon extracellular pigments, gave dark pigmented intracellular extracts from the biomass, indicating that the pigment was also highly concentrated inside the mycelium. These mainly concerned the isolates included in the group “isolates with intense hues”, and in the group “isolates with orange hues” to a lesser extent (Table 3). Thus, isolates appeared with different status and varying capacities, towards pigment production.

### 3.3. Spectrophotometric Characterization of Pigments

**Figure 4:**
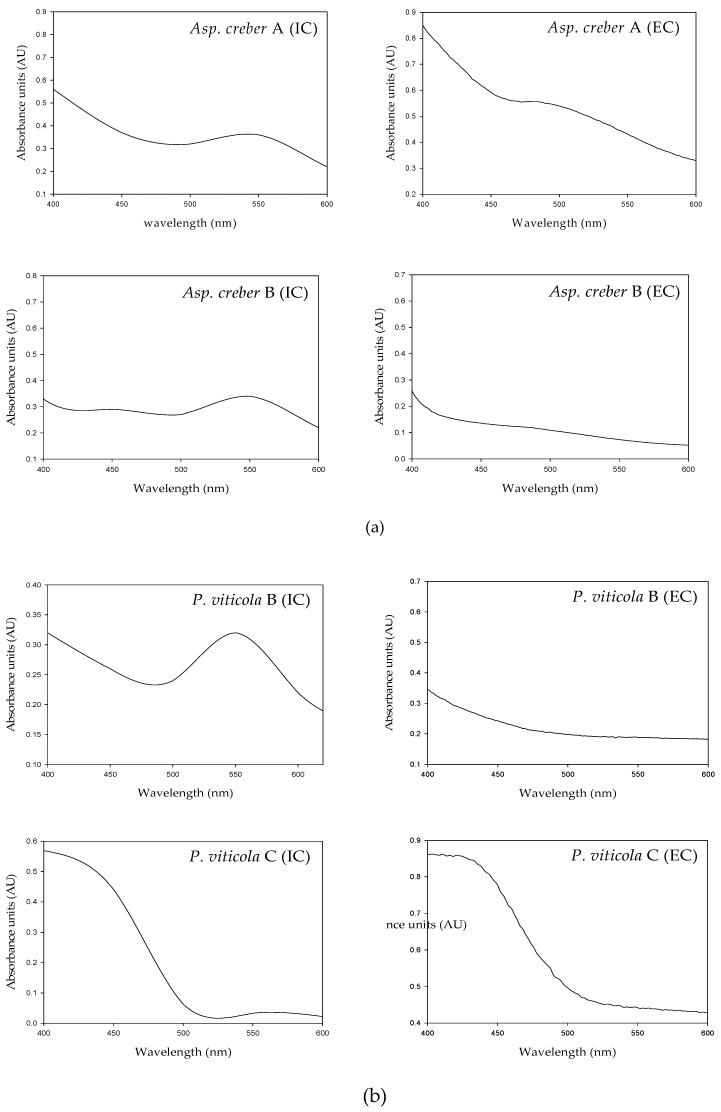

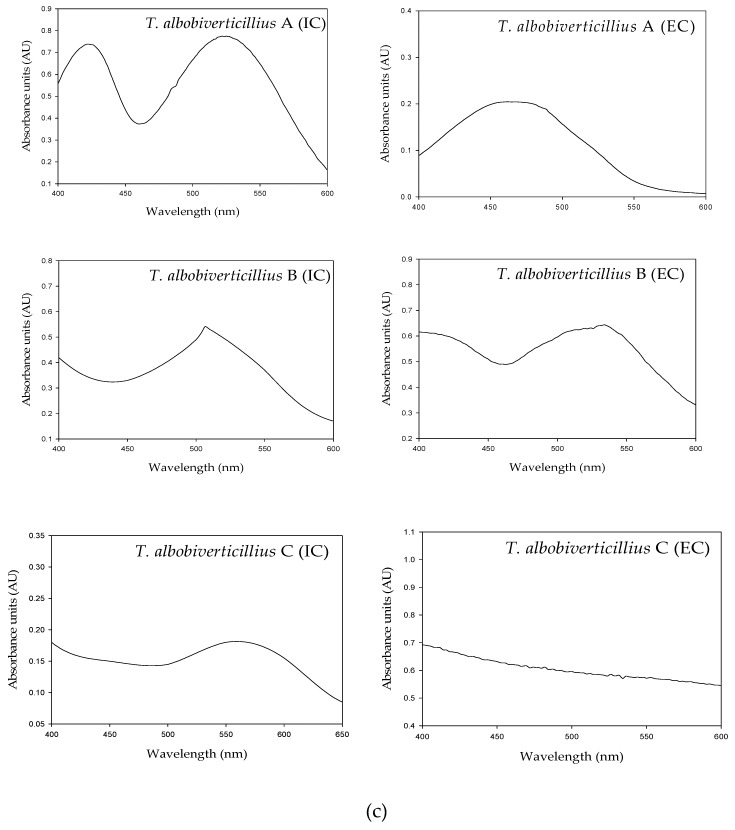
Intracellular (IC) and extracellular (EC) UV-visible spectra of: (**a**) *Aspergillus creber* A and B; (**b**) *Penicillium viticola* B and C; and (**c**) *Talaromyces albobiverticillius* A–C cultures in potato dextrose broth (7 days).

As shown in Figure 4, the absorbance spectra of intra- and extracellular solutions from a single isolate revealed quite similar profiles characterized by a strong absorbance in the UV region and also an area of absorbance in the visible range of wavelengths. The values were principally located in the 400–480 nm area for pale yellow to yellow-orange pigments. The maximal absorbances spread in the 500–550 nm region for red colors.

**Figure 5:**
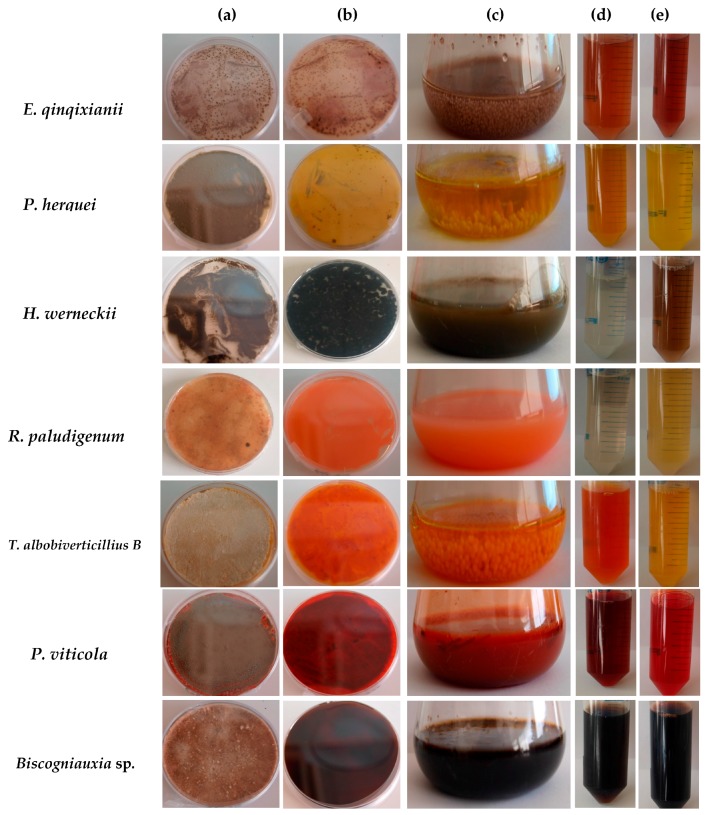
Colors observed in different fungal species: (**a**) obverse face on PDA; (**b**) reverse face on PDA; (**c**) culture in PDB (seven days); (**d**) extract of intracellular pigments (Ethanol/water 50/50) (IC); and (**e**) filtrate from liquid culture (EC).

However, slight variations were noticed between extra- and intracellular liquids: in *A. creber* A as an example, extracellular maximum absorbance was around 470 nm (yellow-orange hue) instead of 550 nm (red shade) for intracellular liquid (Figure 4a). These slight variations however indicate that intra- and extracellular solutions may contain different assortments of colored compounds, in different proportions, resulting in different hues (Figure 5).

Differences were also observed among the spectral profiles of different isolates belonging to the same species. As shown from the intracellular profiles of *A. creber* A and B, *P. viticola* B and C, and *T. albobiverticillius* A–C (Figure 4a–c, respectively, and Table 4), maximal absorbance areas differed in the visible region (510–560 nm for *P. viticola* B and 420–450 nm for *P. viticola* C; and 422–525 nm for *T. albobiverticillius* A, 500 nm for *T. albobiverticillius* B and 520–580 nm for *T. albobiverticillius* C), but, for *A. creber* A and B, the spectra looked similar (Figure 4a,b). Similar variation was stated between the extracellular profiles.

These results clearly imply that isolates from a same species produce and secrete different pigments and therefore have different behavior towards colored compound production.

### 3.4. Evaluation of Intracellular and Extracellular Contents in Pigments

**Figure 6:**
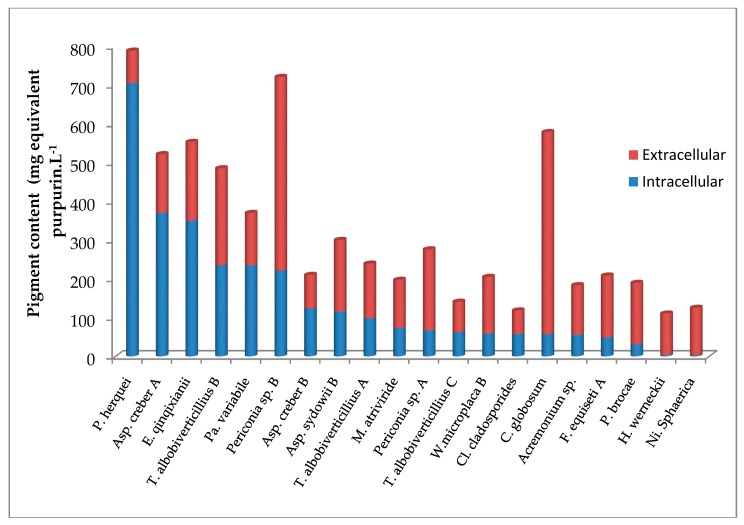
Colored compounds in biomass (intracellular, IC) and culture filtrate (extracellular, EC) for 20 isolates of marine derived fungi isolated around La Réunion Island, in mg eq. purpurin L^−1^ of culture medium (potato dextrose broth, absorbance at 254 nm).

The amount of pigments in IC and EC solutions, expressed in mg eq. Purpurin L^−1^, are presented in Figure 6.

In regard with the diversity of isolates and colored compounds involved in this study, instead of the intensity of the color, the values express the global amount of polyketides compounds produced by each isolate, hues ranging from pale green, light yellow to dark red or maroon.

In the extracellular samples, the maximum amount was produced by *Chaetomium globosum* (521.44 mg equivalent purpurin L^−1^), followed by *Periconia* sp. B (498.39 mg equivalent purpurin L^−1^). For intracellular samples, the maximum levels were measured for *P. herquei*, *A. creber* A, *E. qinqixianii* with 704.55, 371.18 and 350.93 mg equivalent purpurin L^−1^, respectively.

The amount of intracellular content was significantly lower than the one of the extracellular content in this population (*n* = (20,20), *V* = 10, *P* = 6 × 10^−4^). However, looking at each isolate separately, the amount of intracellular pigments was significantly higher than the extracellular one for *P. herquei* (704.55 vs. 84 mg equivalent purpurin L^−1^), and *A. creber* A (371.18 vs. 151.11) and B (125.25 vs. 86.35), and *E. qinqixianii* (350.93 vs. 202.74).

## 4. Discussion

### 4.1. Biodiversity of Marine-Derived Fungi around La Réunion Island

From the sampling in La Réunion Island marine biotopes, 31 different species distributed in 25 genera were identified as pigment producers. The identification of isolates collected in coral reefs and lava flows of La Réunion Island coincides with identifications conducted from various marine environments. Indeed, the majority of the studied fungi, such as those sampled from north of the Indian Ocean, belong to the phylum Ascomycetes. The fungi of the genus *Aspergillus*, particularly *A. sydowii*, are also found in the Caribbean corals (*Porites lobata*), Polynesia, and in sediments off the coast of India [46,47]. *Penicillium citrinum* was isolated from the red algae *Actinotrichia fragilis*, from sponges, and the species was also found on other substrates such as hard substrate or water [48,49,50,51]. The genera *Penicillium, Cladosporium, Chaetomium,* and *Fusarium*, and species *Nigrospora oryzae* and *Hortea werneckii*, have been identified in marine sediments collected at different depths in the central basin of the Indian Ocean and considered to be coral pathogens [8,52]. Fungi, namely *Alternaria* sp*.*, *Acremonium* sp. and *Rhodotorula mucilaginosa*, were isolated from salt lakes in Antarctica, as were *P. chrysogenum* and *P. crustosum* [53]*. Rhodotorula mucilaginosa* was also found in the sediments of central Indian basin [52].

The diversity within the isolated fungal population was crucial while comparing the ability of pigment production [54]. However, in our samples, the highest diversity of pigmented fungi was revealed from the water column (13 species) and from hard substrates (limestone or lava flow) (11 species). If the water column can be suspected of carrying a multitude of fungal propagules originating from terrestrial environments, hard substrates are probably more representative of marine and marine-derived biodiversity.

Our study demonstrates that the living coral *Pocillopora* sp. shelters fungi from the genera *Aspergillus* (*A. creber*, *A. sydowii* and *Eurotium amstellodami*) and *Penicillium* (*P. viticola*), as well as *Hypocrea koningii* and *Acremonium* sp. Widely disseminated on land, this mainly saprophytic genus *Acremonium* sp., has already been isolated from marine environments (sea fans, sea water, sea cucumbers, and intertidal sediment samples) [55,56,57]. These fungi, were extracted from the inner parts of the coral structure. They are then supposed to be at least endophytic species for this coral genus.

The coral rubbles (dead part of corals) from our samples contained colored *Penicillium* (*P. herquei*) or related species *T. albobiverticillius* as well as an isolate of *Chaetomium globosum*. *Chaetomium globosum* is a common fungal species from soil and environment.

Most of the fungi we identified can also be found on land, in soil, on plants or insects, but some of them have rarely been isolated from marine environments such as *Whalleya microplaca, Biscogniauxia* sp., *Paraconiothyrium variabile*, *Myrothecium atroviride*, *Nectria haematococca*, *Peyronellaea glomerata*, *Epicoccum sorghi, Sporisorium exsertum* and *Periconia* sp.. From our study, the genera *Aspergillus* and *Penicillium* or the close ones such as *Talaromyces, Emericella* or *Eurotium* (from the Trichocomaceae family) are much more diverse than others in these tropical marine biotopes (12 different species), and are represented in several types of samples and locations. These aerobic and xerophilic species are well-known for populating dry and/or salty biotopes. However, their ability to subsist or develop underwater, with widely varying oxygenation conditions is less known. These cosmopolitan fungi are well-known to produce a wide range of secondary metabolites such as polyketide-based pigments in solid and liquid cultures. Overall, in our study, some fungal species (*T. albobiverticillius* or *N. haematococca*) were identified from different types of samples in the same area. Some others (*A. sydowii*, *P. viticola*) appeared in separate locations. These fungi found in several sample types and/or in different locations may be considered as frequent in marine environment around La Réunion Island.

### 4.2. Qualitative Aspect of the Pigment Production

For marine-derived isolates, two statuses lead to particular behaviors and products: the challenge of facing unusual living conditions (exogenous fungi) and the use of specific procedures naturally adapted to the marine niches (for instance fungal endophytes of marine microorganisms, i.e., indigenous micromycetes, naturally selected by aquatic environments).

Overall, in unusual biotopes (sometimes extreme), the fungal species with pigmented cell walls (in the spores and/or mycelium), are clearly able to tolerate dehydration-hydration cycles or high solar radiations, better than the moniliaceous fungi, whose cells are devoid of pigments. These aromatic compounds, as melanin, sporopollenin (brown product of oxydative polymerization of β-carotene) or cycloleucomelone (terphenylquinone), often show significant antioxidant activities, and are bound to protect the biological structures, giving them an excellent durability and a high potential for survival in hostile environments [58,59].

From the available literature, the microorganisms of the genus *Trichoderma* are frequent in marine environments and some terrestrial strains are able to produce anthraquinone-like compounds [60]. Indeed, isolates of the family Hypocreaceae (*Trichoderma, Hypocrea* and *Acremonium*) are also represented in our study and exhibit orange to purple hues*.* Some strains of the common soil fungus *Cladosporium cladosporioides*, also isolated from our samples with green shades, have already been studied for their production of intracellular melanin [61].

The most important colored compounds produced by *Aspergillus* and *Emericella* species are respectively, hydroxyanthraquinones and azaphilone pigments, exhibiting a very wide range of hues. Furthermore, *A. sydowii* and *Eurotium amstelodami* isolated from La Réunion Island showed red and yellow colors respectively, as produced by their terrestrial counterparts [62].

*Penicillium* species and related ones seem to adjust easily to multiple conditions and to be a source of original compounds as they appear among the most chemically inventive fungi. In *Penicillium* and *Talaromyces* species, polyketide-based pigments are also very common, and, particularly, the azaphilones, such as the derivatives of monascorubrin and rubropunctatin [63]. *Monascus*-like azaphilone pigments such as *N*-glutarylmonascorubramine, *N*-glutarylrubropunctamine, monascorubramine homologues PP-V [(10Z)-12-carboxyl-monascorubramine] and PP-R [(10Z)-7-(2-hydroxyethyl)-monascorubramine] are frequently identified in their cultures [64,65]. However the commercial production of red anthraquinoid pigments (Arpink Red™, Natural Red™) has already been carried out with *P. oxalicum var. armeniaca* [1]. The most common hues produced by both genera include yellow, red, orange and reddish-brown. Nevertheless, it was found that the yellow pigments seem predominant in most of the *Penicillium* species, while *Talaromyces* species mainly produce red pigments with few synthesizing yellow compounds of azaphilone series [66]. The colored molecules sometimes demonstrate mycotoxic activities such as rubratoxins A and B, rugulovasins and luteoskyrin [67].

Some strains of the widespread *Acremonium* sp. produce the yellow oosporein (chaetomidin) (biquinone, benzoquinone) and also some toxic compounds as diterpene glycosides [68].

*Chaetomium globosum*, isolated from the coral rubbles biosynthesizes maroon pigments in the culture conditions of our experiment. Many members of the family produce metabolites with antifungal properties. *C. globosum* is already known to biosynthesize yellow azaphilones named chaetoviridins (A–D), antifungal compounds involved in the induction of chlamydospores-like cells [69]. It also produces nitrogenous azaphilones (4′-epi-*N*-2-hydroxyethyl-azachaetoviridin A, and *N*-2-butyric-azochaetoviridin E) and isochromophilone XIII, with orange to red hues. Some strains generate pigmented chaetoglobins, chaetoglobosins, chaetomugilins, and seco-chaetomugilins, while others can secrete a purple pigment called cochliodinol [70,71,72,73].

Associated with lava flows, *Fusarium equiseti* belongs to a group of widespread plant pathogens, but marine-derived *Fusarium* strains are also frequent in mangroves or associated with marine organisms. These are already known to produce original colored anthraquinoid compounds (5-acetyl-2-methoxy-1,4,6-trihydroxy-anthraquinone;6,8-dimethoxy-1-methyl-2-(3-oxobutyl)-anthraquinone and fusaquinones) [19]. Among the *Fusarium* secondary metabolites, numerous polyketide pigments have already been identified, such as naphthoquinone pigments which are the most abundant (bikaverin, nor-bikaverin, javanicin, anhydrojavanicin, fusarubin, anhydrofusarubin, bostrycoidin, and novarubin) and the hydroxyanthraquinones emodin, physcion, dermolutein, chrysophanol, erythroglaucin, dermocybin, dermorubin, tritisporin, cynodontin, helminthosporin or aurofusarin (review in [19,21]). All these molecules develop a palette of colors, ranging from yellow to purple or brown. Some species are also able to produce orange carotenoids (neurosporaxanthin by *F. fujikuroi*) [74]. The putative carcinogen, fusarin C, apicidin F, fujikurins, the perithecal pigments fusarubins as well as the mycelial pigment bikaverin are also produced in the family.

From our work, *Periconia* sp. A isolate produced an impressive violet hue in PDB culture. *Periconia* is a cosmopolitan genus, often found in soil, and decaying herbs and forages. Some *Periconia* strains were nevertheless identified from marine environments (*P. abyssa* (deep sea), *P. byssoides* (sea slug *Aplysia kurodai*)) [75,76,77]. They attract interest because of the production of promising anti-cancer drugs, such as the carbosugar pericosine A. Some strains may produce an unidentified hepatoxin.

### 4.3. Quantitative Aspect of Pigment Production

As a promising factor, several of the marine-derived fungi isolated in this study had the ability to grow and biosynthesize pigments in unsalted synthetic conditions (e.g., Czapek Dox medium, PDB). During the period of fermentation, the pigment production started between Day 1 and Day 4 for the majority of isolates such as *Aspergillus, Eurotium*, *Fusarium*, *Nigrospora*, *Pencillium* and *Talaromyces*. For some fungi, the detection of the pigment production was notably delayed (e.g., *Acremonium, Epicoccum*, or *Myrothecium*). This might be due to the low level of pigment producing ability of the fungi or due to unfavorable environmental conditions for pigment production such as pH, temperature, nutrient sources, osmolarity and illumination conditions [78].

Considering the visual observation of pigment color in flasks and the respective UV-visible spectra, fungi belonging to the same species may produce different colored mixtures (e.g., *Aspergillus creber* A and B or *Talaromyces alboverticillius* A–C). They may then belong to different varieties and thus produce pigments of distinct natures. The slight variations observed between intra- and extra-cellular solutions also indicate that the solutions may contain different assortments of colored compounds, in different proportions, resulting in different hues.

From these findings, it is understood that a higher quantity of pigments has been mainly purified from extracellular filtrates in a significant manner (11/21 isolates). In our experimental conditions, the maximum pigment production was obtained in the extracellular samples for *C. globosum* and *Periconia* sp*. B*. On average, the values measured in the cells were significantly lower; indicating that pigments secretion in the liquid medium seems a widespread behavior in the conditions of the experiment. Only the isolate *P. herquei* had a very high level of intracellular pigment biosynthesis (704.55 mg equivalent purpurin L^−1^). Nevertheless, for high intracellular pigment production from biomass, *A. creber* A and *E. qinqixianii* present a true production potential. On the other hand, the extraction of intracellular colored compounds appeared sometimes not completely effective. The fungal biomass was still colored even after extraction. The efficiency of the extraction process could probably be improved to recover higher pigment quantities from intracellular samples [79].

This work highlights different behaviors of fungal isolates towards the secretion of colored molecules compared to internal storage. Anyway, the production of secondary metabolites often occurs after fungal growth has ceased, as a result of nutrient limitation coupled with excess carbon availability. This makes it possible to manipulate their formation [80,81].

## 5. Conclusions

Marine and marine-derived fungi are promising resources for the production of new metabolites of interest, and, among them, pigments are attractive [82,83,84]. The potential of marine-derived microorganisms to produce unique and original molecules may come from specific metabolic or genetic adaptation appearing to meet very specific combinations of physical and chemical parameters (high salinity, low O_2_ penetration, low temperature, limited light access and high pressure). Based on this statement**,** our study explores, for the first time, the biodiversity of fungi from marine environments around La Réunion Island, Indian Ocean, along with the ability of the isolates to produce pigments. The potentiality of these marine derived isolates to secrete pigments or to concentrate colored compounds inside the cells was highlighted. Several isolates collected from lava flows, hard substrates sediments and corals (living or dead) turned out to be the interesting producers of intense colors on PDA culture medium. The main types identified, *Aspergillus*, *Penicillium* and related genera, are also found in other marine regions (such as Polynesia or along the coast of India). However, a great biodiversity (31 species) emphasizes the range of possible hues and molecules susceptible to be isolated. The majority of the isolates, probably marine optional, may also be able to grow in synthetic media, devoid of sea salts and may show the competence of producing pigments in an industrial scale. The most promising pigmented products, probably of intense red or purple hues, which seem to consist in mixtures, will be subjected to purification and further analyses by analytical techniques such as liquid chromatography–mass spectrometry/time-of-flight (LC-MS/TOF) and Nuclear Magnetic Resonance (NMR). The interesting isolates will also be subjected to further analyses to determine their ability as antibiotics or for enzyme production.

## Figures and Tables

**Table 1:** PCR amplification and the sequencing primers used for the identification of fungal isolates.

Primers	Direction	Sequences (5’→ 3’)	Note	Hybrid. T °C	Refs.
ITS1-F_KYO2	Forward	TAGAGGAAGTAAAAGTCGTAA	Small sub-unit, ITS 1, 5.8S, ITS 2, Largest sub unit rDNA	56	[36]
ITS2_KYO2	Reverse	TTYRCTRCGTTCTTCATC		47	
ITS3_KYO2	Forward	GATGAAGAACGYAGYRAA		47	
ITS 1	Forward	TCCGTAGGTGAACCTGCGG		55	[39]
ITS 2	Reverse	GCTGCGTTCTTCATCGATGC		55	
ITS 3	Forward	GCATCGATGAAGAACGCAGC		55	
ITS 4	Reverse	TCCTCCGCTTATTGATATGC		55	
V9G	Forward	TTACGTCCCTGCCCTTTGTA	Large sub unit D1/D2 for basidiomycetous yeast	55	[38]
LR3	Reverse	TGACCATTACGCCAGCATCC		57	
Cmd 5	Forward	CCGAGTACAAGGARGCCTTC	Calmodulin, specific for *Aspergillus*	52	
Cmd 6	Reverse	CCGATRGAGGTCATRACGTGG		52	
T 10	Forward	ACGATAGGTTCACCTCCAGAC	β- tubulin, specific for *Penicillium*	55	[38]
Bt2b	Reverse	ACCCTCAGTGTAGTGACCCTTGGC		55	
EF1-728F	Forward	CATCGAGAAGTTCGAGAAGG	Elongation factor 1 for *Trichoderma*	55	[38]
TEF1-LLErev	Reverse	AACTTGCAGGCAATGTGG		55	

**Table 2:** Fungal isolates from La Réunion Island marine biotopes from different sample types and sampling sites: Trou d’Eau (TDE); Planch’ Alizé (PA); Lava flow corresponds to 1977 lava flow in Sainte Rose/Tremblet area.

Family	Fungal Species	Sampling Site	Gene Accession Number
***Water Bodies***			
**Davidiellaceae**	*Cladosporium Cladosporioides*	Lava flow (−70 m)	JF949719.1
**Didymellaceae**	*Peyronellaea glomerata* (syn: *Phoma glomerata*)	Lava flow (−70 m)	JQ936163.1
**Nectriaceae**	*Nectria haematococca* A	Lava flow (−70 m)	XM_003053163.1
**Pleosporales Incertae Sedis**	*Periconia* sp. A	Lava flow (−70 m)	HQ608027.1
	*Periconia* sp. B	Lava flow (−70 m)	HQ608027.1
**Sporidiobolaceae**	*Rhodosporidium paludigenum*	Lava flow (−70 m)	AF444493.1
**Stachybotryaceae**	*Myrothecium atroviride*	Lava flow (−70 m)	AJ302002.1
**Teratospheriaceae**	*Hortaea werneckii (syn: Cladosporium werneckii*)	Lava flow (−70 m)	JN997372.1
**Trichocomaceae**	*Aspergillus sydowii* B	Lava flow (−70 m)	KC253961.1
	*Emericella qinqixianii*	TDE outer slope	AB249008.1
	*Penicillium brocae NRRL 32599*	TDE outer slope	DQ123642.1
	*Penicillium viticola* B	TDE inner reef	AB606414.1
	*Talaromyces rotundus*	TDE inner reef	EU497950.1
	*Talaromyces verruculosus*	PA inner reef	KC416631.1
**Wallemiaceae**	*Wallemia sebi*	Lava flow (−70 m)	Morphological Identification
***Living Coral*** *Pocillopora sp.*			
**Hypocreaceae**	*Acremonium* sp.	PA inner reef	FJ770373.1
	*Hypocrea koningii*	TDE inner reef	JX174420.1
**Trichocomaceae**	*Aspergillus creber* A	TDE inner reef	JN854049.1
	*Aspergillus creber* B	TDE inner reef	JN854049.1
	*Aspergillus sydowii* A	PA inner reef	JN854052.1
	*Eurotium amstelodami*	TDE outer slope	FR727111.1
	*Penicillium viticola* C	PA inner reef	AB606414.1
***Coral Rubbles***			
**Chaetomiaceae**	*Chaetomium globosum or Chaetomium murorum*	TDE outer slope	JN209898.1
**Trichocomaceae**	*Penicillium herquei*	TDE outer slope	JN246042.1
	*Talaromyces albobiverticillius* B	TDE outer slope	JN899313.1
	*Talaromyces albobiverticillius* C	TDE outer slope	JN899313.1
***Hard Substrate/Rock Substrate***			
**Nectriaceae**	*Fusarium equiseti* A	Lava flow (−25 m)	JQ936153.1
	*Fusarium equiseti* B	Lava flow (−25 m)	JF311925.1
	*Fusarium equiseti* C	Lava flow (−25 m)	JQ936153.1
	*Nectria haematococca* B	Lava flow (−25 m)	XM_003053163.1
**Pleosporaceae**	*Epicoccum sorghi* (syn: *Phoma sorghina; Peyronellaea stemphylioides*)	Lava flow (−25 m)	KC106717.1
**Sordariomycetes**	*Nigrospora sphaerica* (or Env. sample from marine air*)*	TDE outer slope	KC505176.1
**Sporidiobolaceae**	*Rhodotorula mucilaginosa*	TDE outer slope	KC515367.1
**Trichocomaceae**	*Penicillium citrinum*	Lava flow (−25 m)	EU030332.1
	*Penicillium viticola* A	Lava flow (−25 m)	AB606414.1
**Ustilaginaceae**	*Sporisorium exsertum*	TDE outer slope	JN367293.1
**Xylariaceae**	*Biscogniauxia* sp.	PA inner reef	FJ884075.1
	*Whalleya microplaca* A	TDE outer slope	JQ760548.1
	*Sordariomycete* (or *Whalleya microplaca* B)	TDE outer slope	FJ416301.1
***Sediments***			
**Didymosphaeriaceae**	*Paraconiothyrium variabile*	TDE outer slope	JQ936271.1
**Hypocreaceae**	*Trichoderma atroviride*	TDE outer slope	KC008065.1
**Trichocomaceae**	*Talaromyces albobiverticillius* A	TDE outer slope	JN899313.1

**Table 3:** Dominant colors of culture broth ^1^, extracellular (EC) ^2^ and intracellular (IC) ^3^ pigments from fungal isolates.

Fungal Isolates	Approximate Hues			Fungal Isolates		Approximate Hues		
**Isolate*s with Intense Hues (Purple/Red/Maroon)***				***Isolates with Orange Hues***				
	**Broth**	***EC***	***IC***		**Broth**		***EC***	***IC***
*Acremonium* sp.				*Penicillium viticola* A				
*Talaromyces albobiverticillius* A				*Penicillium viticola* B				
*Talaromyces albobiverticillius* B				*Epicoccum sorghi*				
*Talaromyces albobiverticillius* C				*Penicillium brocae NRRL 32599*				
*Aspergillus sydowii* A				*Penicillium herquei*				
*Aspergillus creber* A				*Aspergillus sydowii* B				
*Aspergillus creber* B				*Chaetomium globosum* or *C. murorum*				
*Emericella qinqixianii*				*Penicillium viticola* C				
*Trichoderma atroviride*				*Penicillium citrinum*				
*Biscogniauxia* sp.				*Hypocrea koningii*				
*Paraconiothyrium variabile*								
*Myrothecium atroviride*								
***Isolates with Yellow Hues***				***Isolates with Green/Brown Hues***				
	**Broth**	***EC***	***IC***		**Broth**		***EC***	***IC***
*Peyronellaea glomerata*				*Talaromyces verruculosus*				
*Eurotium amstelodami*				*Talaromyces rotundus*				
*Rhodosporidium paludigenum*				*Wallemia sebi*				
*Periconia* sp. A				*Sporisorium exertum*				
*Periconia* sp. B				*Hortea werneckii*				
*Rhodotorula mucilaginosa*				*Whalleya microplaca* A				
*Fusarium equiseti* A				*Whalleya microplaca* B				
*Fusarium equiseti* B				*Nigrospora sphaerica or Env. sample*				
*Fusarium equiseti* C				*from marine air*				
*Nectria haematococca* A				*Cladosporium cladosporioïdes*				
*Nectria haematococca* B								

^1^ Culture broth: mycelium + liquid medium; ^2^ EC: filtrate from liquid culture medium; ^3^ IC: intracellular extract of fungal pigments.

**Table 4:** Summary of main peaks (λ_max_) noticed in 10-days old culture of *Talaromyces albobiverticillius* isolates A–C cultivated in liquid medium (potato dextrose broth).

*T. albobiverticillius*	Sample	Peaks in the UV Region (nm)		Peaks in the Visible Region (nm)	
		200–250	250–300	300–400	>400
A	IC	235	286	362	422, 425, 511, 525
	EC		265	365	458, 469.8, 480
B	IC	232	268, 292		410, 440, 460, 500
	EC		288		412, 524, 532
C	IC	222	283	385	520–580
	EC		283	370, 385	436

## Supplementary Materials

The following are available online at www.mdpi.com/2309-608X/3/3/36/s1.
