# Supplementary material for: Biodiversity of Pigmented Fungi Isolated from Marine Environment in La Réunion Island, Indian Ocean: New Resources for Colored Metabolites
Source: J Fungi (Basel). 2017 Jul 2;3(3):36. doi: 10.3390/jof3030036 (PMC5715948; doi:10.3390/jof3030036)
Supplement: Supplementary file 1 [file jof-03-00036-s001.pdf]

Biodiversity of Pigmented Fungi Isolated from Marine Environment in La Réunion Island, Indian Ocean: New Resources for Colored Metabolites

Mireille Fouillaud *, Mekala Venkatachalam, Melissa Llorente, Helene Magalon, Pascale Cuet and Laurent Dufossé

Appendix A

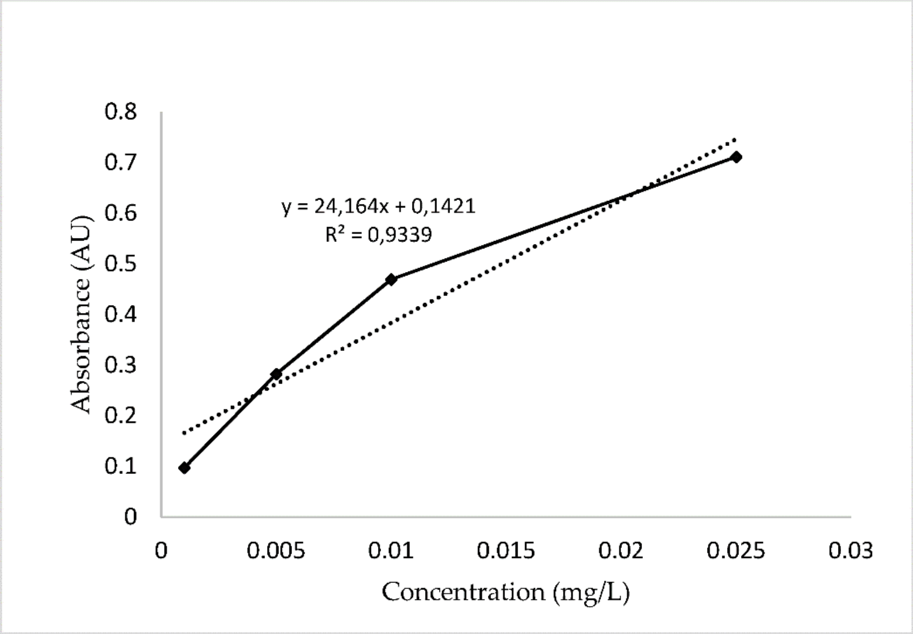

Figure S1. Standard curve for purpurin at 254 nm (- raw data; ….. linear regression)
